# Frequency of urinary pesticides in children: a scoping review

**DOI:** 10.3389/fpubh.2023.1227337

**Published:** 2023-08-29

**Authors:** Horacio Guzman-Torres, Elena Sandoval-Pinto, Rosa Cremades, Adrián Ramírez-de-Arellano, Mariana García-Gutiérrez, Felipe Lozano-Kasten, Erick Sierra-Díaz

**Affiliations:** ^1^Departamento de Salud Pública, Centro Universitario en Ciencias de la Salud, Universidad de Guadalajara, Guadalajara, Jalisco, Mexico; ^2^Departamento de Biología Celular y Molecular, Centro Universitario de Ciencias Biológico Agropecuarias, Universidad de Guadalajara, Guadalajara, Jalisco, Mexico; ^3^Departamento de Microbiología y Parasitología, Centro Universitario en Ciencias de la Salud, Universidad de Guadalajara, Guadalajara, Jalisco, Mexico; ^4^Instituto de Investigación en Ciencias Biomédicas, Centro Universitario de Ciencias de la Salud, Universidad de Guadalajara, Guadalajara, Mexico; ^5^Centro Metropolitano de Atención de la Diabetes Tipo 1, OPD Servicios de Salud, Secretaría de Salud Jalisco, Guadalajara, Jalisco, Mexico; ^6^Departamentos de Clínicas Quirúrgicas y Salud Pública, Centro Universitario de Ciencias de la Salud, Universidad de Guadalajara, Guadalajara, Mexico; ^7^División de Epidemiología, UMAE Hospital de Especialidades Centro Médico Nacional de Occidente del IMSS, Guadalajara, Mexico

**Keywords:** urinary pesticide, levels, children, exposure, glyphosate, organic diet

## Abstract

Pesticides are any mix of ingredients and substances used to eliminate or control unwanted vegetable or animal species recognized as plagues. Its use has been discussed in research due to the scarcity of strong scientific evidence about its health effects. International literature is still insufficient to establish a global recommendation through public policy. This study aims to explore international evidence of the presence of pesticides in urine samples from children and their effects on health through a scoping review based on the methodology described by Arksey and O‘Malley. The number of articles resulting from the keyword combination was 454, and a total of 93 manuscripts were included in the results and 22 were complementary. Keywords included in the search were: urinary, pesticide, children, and childhood. Children are exposed to pesticide residues through a fruit and vegetable intake environment and household insecticide use. Behavioral effects of neural damage, diabetes, obesity, and pulmonary function are health outcomes for children that are commonly studied. Gas and liquid chromatography-tandem mass spectrometry methods are used predominantly for metabolite-pesticide detection in urine samples. Dialkylphosphates (DAP) are common in organophosphate (OP) metabolite studies. First-morning spot samples are recommended to most accurately characterize OP dose in children. International evidence in PubMed supports that organic diets in children are successful interventions that decrease the urinary levels of pesticides. Several urinary pesticide studies were found throughout the world's population. However, there is a knowledge gap that is important to address (public policy), due to farming activities that are predominant in these territories.

## 1. Introduction

Human health is described as the essential condition to sustain human beings. Ever since the *Homo sapiens* era, the cognitive capacity of human beings has been an important factor in our role within the evolutionary tree and has also functioned as a key to guaranteeing the stability of human life. Unfortunately, some of these human capacities have harmed other living species. The unlimited exploitation of natural resources to derive economic profit has brought about negative consequences in the environment, and some of them remain unknown (1).

One such way of causing environmental damage is the use of pesticides. Pesticides are mixtures of substances that are used to eliminate or control undesirable plant or animal species, and their effects behave in a specific way. There are three broad classifications according to their target population: herbicides for plant control, insecticides for the management of bugs and insects, and fungicides that are used to eliminate fungi (2).

Several types of pesticides have been used, and in general are accepted, based on their role in agricultural activities and economic development in several countries. By using substances such as insecticides, herbicides, and fungicides, it is possible to reduce damage to agricultural products, thereby improving quality, and in some cases, the yield (3). Farming activities have not always been easy. In the past, crops suffered from pests and diseases which resulted in a decreased production of vegetables and fruits. Today, through the use of pesticides, the loss of vegetables and fruits has decreased by 78 and 54% respectively (3, 4).

However, not everything is positive with regard to the use of pesticides. Several authors have reported the harmful health effects of pesticide exposure in humans and other living species (pollinators), which is considered a global concern and a public health issue (5–8). Pesticide exposure has been related to several chronic diseases such as cancer, birth defects, neurodegenerative disorders, and others. Cancer and neurodegenerative damage are the most commonly studied cases (9–12).

In 2015, the International Agency for Research on Cancer (IARC), the cancer agency of the World Health Organization (WHO), declared glyphosate a potential carcinogen. Indeed, glyphosate is the most common pesticide used around the world. Unfortunately, this genotoxic herbicide is only one of several toxic compounds used in agricultural activities (13). Several damage mechanisms to human health related to pesticide use are reported in international literature.

A disturbance in cellular homeostasis is followed by disruptions in ion channels, receptors, and enzymes, and can result in the changing of some metabolic pathways and mechanisms (6). Neurodegenerative damage has been associated with reactive oxygen metabolism and the disruption of mitochondrial bioenergetics. Some polymorphisms are associated with the risk of Parkinson's disease after exposure to pesticide-metabolizing enzymes (14). Other studies reported that exposure to pesticides such as paraquat increases the risk of neuroblastoma due to overexpression of α-synuclein (mutated A53T) (15). Glyphosate exposure showed no overexpression of the protein. Other chemical compounds increase the protein levels which are associated with activity in melanoma cell lines (SK-MEL-2) and higher levels of A53T mutated protein related to neurodegenerative damage (15).

In some regions of Europe, carbamates, organophosphates (OPs) (acetylcholinesterase inhibitors), and organochlorines were associated with domestic animal poisoning. Banned pesticides were reported in the north of Italy (carbofuran and methamidophos) (16). The illegal use of pesticides is not limited to some regions. The problem has spread throughout the world, in both developed and developing countries (16–19).

The use of pesticides has a long history and has been a talking point among researchers, due to the scarcity of strong scientific evidence regarding their health effects. The international literature contains many articles in relation to this topic. Despite this amount of knowledge, it is still insufficient for establishing a global recommendation for public policy. Nevertheless, the need for research concerning pesticides is still relevant in countries such as Mexico, as well as for those in Latin American areas where their usage remains a common human practice. The question for this scoping review focuses on the following: What has been studied regarding the presence of pesticides in the urine of children?

This study aims to explore the international evidence of the presence of pesticides in urine samples from children and their effects on health through a scoping review methodology.

## 2. Methods

Based on the methodology described by Arksey and O‘Malley (20), before an extensive review of information, the question for the literature review was defined using keywords included in MeSH terms. The only source of information was PubMed. The screening was performed by a work team using different word combinations. Filters were used in order to limit the number of hints and to obtain the most specific manuscripts regarding the topic. The keywords included in PubMed were: urinary, pesticide, children, and childhood, but not adolescents. The number of articles resulting from the keyword combination was 454. Boolean operators, AND and NOT, were included among the keywords, and the number of hints is described in Table 1.

**Table 1:** Keyword combination and PubMed results.

**Keywords and boolean operators**	**Number of hints**	**Final selection**
Urinary pesticides AND children	200	88
Urinary pesticides AND children NOT adolescents	134	0
Urinary pesticides AND childhood	28	0
Pesticides AND children health NOT adults	92	5
Total	454	93

**Figure 1:**
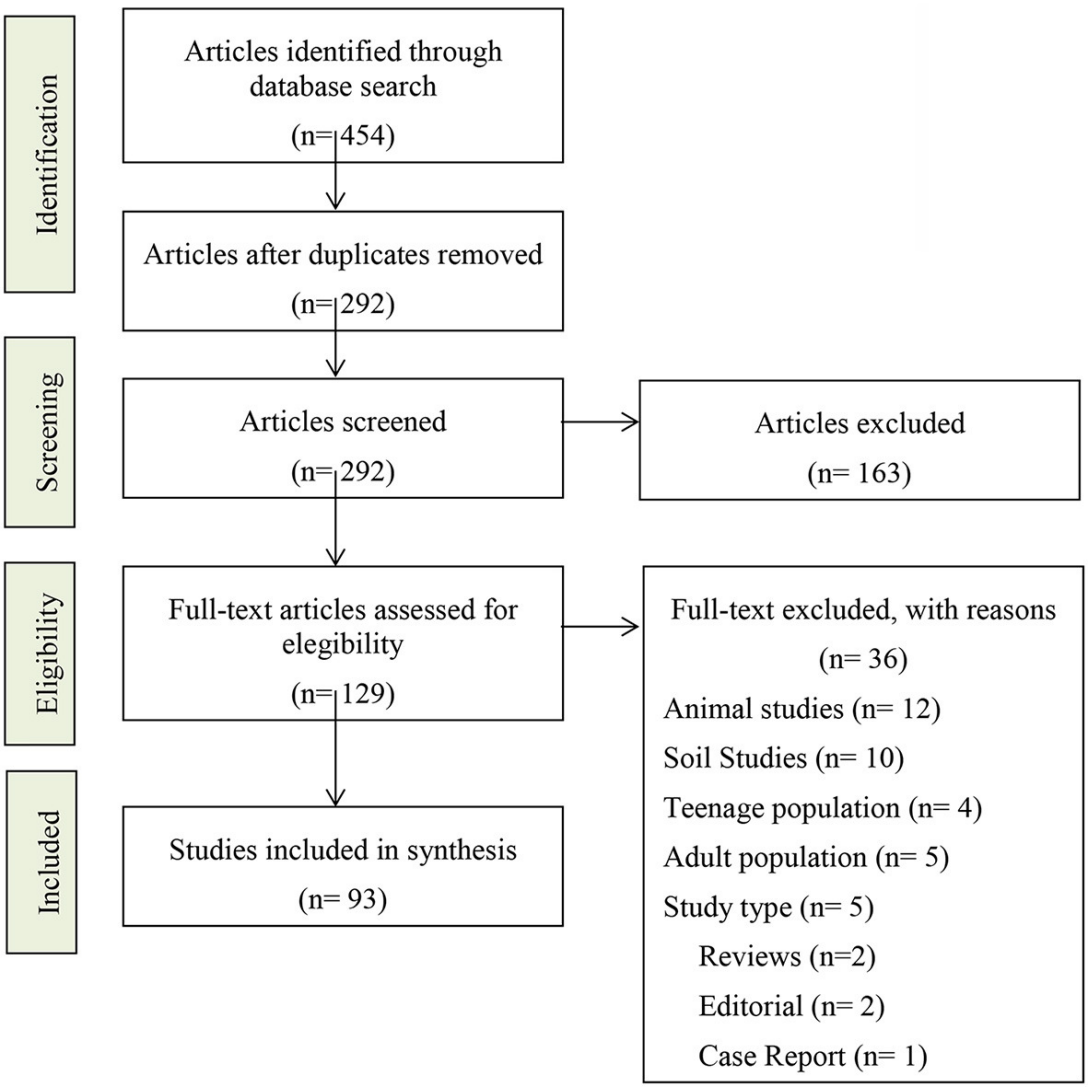
PRISMA ScR chart.

A total of 93 articles were selected for the results section, and 22 were used for complementary sections. The basic selection criteria were: English language, quantitative empirical manuscripts, and applied research in children, published during the last 10 years. Research in adults and adolescents, prenatal-only pesticide research, qualitative research, reviews, and clinical cases were excluded from this study. All the included articles were full text. Articles with only a summary were not considered. A review and selection of the articles were performed over a 6-month period (2021–2022), and after several suggestions, new information was added from May to July 2023. The selected articles were reviewed and mapped by main topics such as diet, geography, occupation, and health facts. After being divided into topics, the articles were systematized in an excerpt matrix (worksheet) that included: the lead author, year and place, study design, sample, variables, statistical analysis, and results (Supplementary Table 1). This process is outlined in the PRISMA-ScR chart (Figure 1).

## 3. Results

Pesticides are used in all countries. Selected articles included countries such as Canada, the United States, Mexico, Costa Rica, Colombia, Brazil, Chile, Argentina, Spain, France, Germany, Italy, the United Kingdom, Norway, Greece, Slovenia, Lithuania, South Africa, India, Taiwan, Iran, Thailand, Israel, Malaysia, Japan, China, South Korea, Saudi Arabia, Vietnam, Cyprus, and Australia (Supplementary Table 1).

The most common methods used to measure pesticides were: high-pressure liquid chromatography spectrometry (HPLC-MS/MS), isotope-dilution gas chromatography–tandem mass spectrometry (GC–MS/MS), and gas chromatography/mass spectrometry (GC/MS) (Supplementary Table 1).

The pesticides most commonly reported in the selected articles were: N-desmethyl-acetamiprid, pyrethroids, organophosphates, DAP, 3-PBA, permethrin, TCPY, glyphosate, CPS, and other pesticide compounds.

Children's health is related to factors such as diet. The ingestion of food constitutes a route of exposure to pollution and also a mechanism of risk to health, therefore, the possible presence of chemical residues such as pesticides may be present in the food that children consume (21).

### 3.1. Diet

Some findings point out the relevance of diet in children's exposure to pesticides. In Australia, Li Y. et al. evaluated the concentration of pesticides and their respective trend in the associative model, (*n* = 400, 0–5 years of age). They found a significant increase in concentrations of DETP, TCPY, 4-nitrophenol, and 3-PBA according to age, which suggested that exposure increases after weaning, or as a result of increased dietary intake, mobility, and activity (22). In Greece, Myridakis et al. noted the exposure of preschoolers (*n* = 500) to several contaminants including pesticides. It was observed that the presence of organophosphate levels was linked to food consumption (23). In India, urine samples were evaluated to determine significant changes in exposure between sexes (*n* = 377, 6–15 years of age), in children in a conventional diet. It found that girls had 87% higher levels of DAP pesticides than boys (24).

#### 3.1.1. Organic diet

In a cohort study conducted in the United States of America, within integrated families with children between 4–15 years of age (*n* = 9), it was found that interventions consisting of organic diets significantly decreased the levels of 13 pesticide metabolites and their original compounds corresponding to organophosphates (malathion and chlorpyrifos), neonicotinoids (clothianidin), the herbicide 2,4-D, and pyrethroid insecticides in urine compared to the levels of pesticides that are found at a baseline under the conditions of a conventional diet (25).

Fagan et al. demonstrated that a 6-day intervention based on an organic diet applied to children (*n* = 9, 4–15 years of age) resulted in a decrease in urine glyphosate levels by 71% and its metabolite AMPA by 76% (95% CI) (26). Other authors agree in their findings that an organic diet decreases concentrations of metabolites in organophosphate insecticides and the herbicide 2,4-D in urine levels in children (*n* = 40, 3–6 years of age) (27). Venners et al. evaluated 2,4-D exposure in four municipalities in British Columbia, Canada, in (*n* = 40) households, with at least one child aged 18–72 months. The statistical analysis reported that urinary levels of 2,4-D were lower when more than 50% of the diet was organic. Among other contributions, this study points out that food may be a route of exposure to 2,4-D (28).

A cross-sectional study located in five countries in the European Union concluded that fruit consumption was positively associated with the presence of organophosphate pesticide metabolites in infant urine (*n* = 1,288), while the consumption of organic food was negatively associated with organophosphate metabolites (29). Another study from Norway reported that fruit consumption was the largest factor contributing to a variation in urinary DAP levels. As a result, they concluded that the consumption of organic fruit is the best way to reduce infant and maternal exposure to organophosphate pesticides (30). These studies agreed that organic diets are effective ways to substantially reduce exposure to pesticides, glyphosate herbicides, and organophosphates.

#### 3.1.2. Fruit and vegetable consumption

In 2012, research by Lemke et al. found no clear association between exposure to glyphosate or AMPA and a vegetarian diet or consumption of cereals, pulses, or vegetables, nor could it be identified (31). In a performed cohort design study conducted in the USA, researchers found no associated patterns between dimethyl metabolite levels (DMAP) and the consumption of fruits, vegetables, or apple juice throughout the planting-harvesting seasons, while only significant relationships were found between vegetable consumption during the harvesting season and the presence of levels of metabolite dimethyl (*p* < 0.002) (32). However, in a cross-sectional study in Israel on children (*n* = 103, 4–11 years of age), fruit consumption was associated with higher levels of DAP metabolites in urine, which indicates evidence of exposure to organophosphate pesticides, where some of the children studied were exposed to levels above those considered safe (33). The frequency and method of consumption are also related to pesticide levels in children. In Malaysia, a child population (*n* = 180, age 7–12 years) was studied, in which it was reported that children who frequently consumed apples had four times more risk of detection of pesticides than those who consumed cucumbers less frequently (34). A study performed on a cohort in Spain (*n* = 559, 3–11 years old), concluded that not washing fruit before eating is a factor that can determine the differences in exposure levels (35).

In Australia, a cross-sectional study concerning pesticide exposure in children (*n* = 56, 12.9 months of average age), found by multivariate analysis a positive association between vegetable consumption and the metabolite 3-PBA, while, in general, fruit and vegetable consumption was associated with the presence of total organophosphate metabolites in urine. In this sense, other factors associated with the presence of concentrations of insecticide metabolites were diet, age, mobility, having pets, frequency of pesticide use at home, frequency of hand washing, and the season, where variables such as hand washing and washing fruits and vegetables are modifiable behaviors, therefore, educational interventions are suggested to minimize childhood exposure to insecticides (36).

Thomas et al. found in the toddler population (*n* = 41, 15–18 months of age), that organophosphate flame retardants were detected in 100% of urine samples. The research reported that higher income (>10,000 US dollars annually) is associated with lower levels of 1,3-dicchoro-isopropyl (TDCPP) and triphenyl phosphate (TPP), in addition to isopropylated triphenyl phosphate (ITP). This fact suggests that with higher income, the quality of meals is better, avoiding fast food and other industrialized products such as sausages. Toddlers of mothers with an income of < 10,000 US dollars annually reported higher levels of the three compounds (TTP/TDCPP/ITP) in the urine (7.8, 12.11, and 4.69 ng/mL respectively). They concluded that a diet consisting of fresh food is related to lower levels of organophosphate flame retardants. The authors suggested that the lack of fresh meals and an increase in meat and fish consumption is associated with higher urinary levels of organophosphate flame retardants (37).

### 3.2. Environmental and educational interventions

Pesticide exposure has also been studied as an outcome of human-environmental interactions and work environments since educational interventions have focused on their effectiveness in reducing pesticide exposure.

Galea KS et al. compared biomarkers of urinary concentrations in children (*n* = 24, 4–12 years of age) during pesticide application (roseate events). Approximately 98 and 97% of penconazole and captan concentrations were lower than the Regulatory Exposure Assessment-predicted exposures, respectively. Although a number of the chlorpyrifos and chlormequat spray-related urinary biomarker concentrations were greater than the predictions, the concentrations suggest these were not significantly different from the levels expected had no pesticide spraying occurred (38).

Werthmann et al. found green building practices had no impact on children's pyrethroid urinary concentrations. Further studies with larger sample sizes are needed to confirm these findings (39).

#### 3.2.1. Geographical factors

Several articles concluded that geographical issues are key factors in the risk of pesticide exposure in children, which further illustrates the relevance of a child's environment in this regard. In a cross-sectional study conducted in Japan, the researchers found that childhood exposure to neonicotinoid pesticides by inhalation was not associated with the season of application (spraying) in pine wilt disease control fields; however, the presence of 6 neonicotinoids reflected the high intake of agricultural products (40). In another cross-sectional study conducted in Australia, Heffernan et al. evaluated pesticide exposure from a sample selected from the general population. The urine pools of 100 specimens were analyzed, and it was observed that the highest concentrations of five organophosphate metabolites were found in the youngest and oldest strata. This may be related to age-specific differences such as behavior or physiology. Additionally, it was found that the levels of metabolites of the organophosphate insecticide, chlorpyrifos, were higher than those reported in the USA and Canada. This may be due to differences in the registered applications of pesticides that exist between countries (41).

In Mexico, the presence of pesticides such as glyphosate, metoxuron, and malathion, was detected in 100% of the children from two agricultural communities (urine samples). The study reported a total of 17 pesticides in urine samples (42). In this regard, a cross-sectional maternal-infant study conducted in Slovenia (*n* = 168, 7–8 years of age and *n* = 178 pairs of mothers and their children), authors found that all metabolites of organophosphate pesticides (PNP, 3-PBA) and pyrethroids (TCPY) analyzed in urine samples had the highest concentrations and were discovered in children, which in mothers was also associated with higher concentrations; and even higher levels occurred in children (43). Raherison et al. studied exposure to airborne pesticides *n* = 96 in children who were living in the vicinity of vineyards located in rural areas in France, where the main airborne pesticides found outside its walls were fungicides and insecticides. Children living in rural areas near vineyards are at increased risk of exposure to dithiocarbamates during the summer, while an association was found between urinary concentrations of ETU and symptoms of asthma and rhinitis (44). In a two-year cohort conducted in communities near farms in Chile in 2020, Muñoz-Quezada et al. found that all the children evaluated had more than two metabolites of urinary pesticides, where 3-PBA was the most frequent (45).

In 2021, in the Western Cape, South Africa, Molomo et al. reported pesticide exposure as the sum of three urinary concentrations of DAP phosphate metabolites in a sample of children (*n* = 183, 9–14 years of age). The study further notes that in younger children, living near grape and apple farms was associated with increased urinary DAP concentrations (46). In another cross-sectional study in the USA, pesticide exposures inside and outside the home were analyzed in children (*n* = 1,094, 6–11 years of age), in which it was determined that exposure both inside and outside the home does not affect the levels of any dialkyl phosphates. The group of children always presented higher levels than adolescents and adults (47). In Japan, Yoshida et al. studied children who were exposed (*n* = 132, 6–15 years of age) to environmental local conditions. From the analysis of urinary pesticides and air quality within its walls, it was found that transfluthrin was the most remarkable pyrethroid as an intramural pollutant (48).

Other research examined the characteristics of exposure to imidacloprid (IMI) in urine samples in children living in rural areas (*n* = 247, average age 5 years), and found that younger children tend to be at higher risk of exposure. Inhabitants of areas with orchids were more exposed to IMI to various degrees (49). Ospina et al. determined in urinary pesticide analyses of children (*n* = 505, 3–5 years of age), that an ethnic group comprised of Asians was more likely than non-Asians to have N-desmethyl-acetamiprid concentrations greater than the 95th percentile (50). In light of this, another study evaluated urinary concentrations of 2,4-D in children in central and southern China, *n* = 108, and found a positive correlation between these concentrations and the biomarker of oxidative stress 8-OHdG in young children (51).

A cross-sectional study in South Korea reported that 10% of the child population, (*n* = 70, 6–12 years of age) with higher urinary levels of 3-PBA tended to be girls who were younger than 9 years of age, who lived in a rural area in an apartment, and usually had higher concentrations of urinary 3-PBA than those in other countries (52). Lehmler et al. characterized exposure to urinary pyrethroid pesticides and evaluated demographic and social factors of infants (*n* = 2,295). It was determined that age, gender, race/ethnicity, and PIR were associated with levels of 3-PBA (53).

In a cohort study carried out in the United Kingdom (*n* = 22, average age of 8 years), researchers point out that they found no evidence indicative of urinary pesticide biomarkers in addition to chlorpyrifos, resulting from roseling or the application of pesticides per season, suggesting that there are other responsible sources in addition to pesticide roseling, of the relatively low urinary biomarkers of pesticide detected (54). Other research studied the presence of pesticides in the general population (*n* = 322, 1–8 years of age), in which they determined organophosphate components in 77% of the total urine samples analyzed. It found that the intake of chlorpyrifos is higher in the populations of Vietnam, Greece, India, China, and Korea (≥9.6 μg/day) than those estimated for other countries (< 5 μg/day). Similarly, the daily intake of parathion was found to be higher in China, India, and Korea, than estimated in other countries (5.7–9.3 μg/day) (55).

#### 3.2.2. Occupation of relatives

Another relevant factor is the activities that relatives perform and their impact on children's exposure. A study (*n* = 180, age 7–12 years) concluded that children whose parents' occupation was related to agricultural work had a 3 times higher risk of pesticide detection than those who have a non-agricultural parent (34). In France, a maternal and infant cohort study reported that the variable of the child's father being occupationally exposed to pesticides means that he is three times more likely to have higher concentrations of 3-PBA, while the household dust content is correlated with insecticide use, higher mean concentrations of permethrin (0.3–1.3 μg/g), and an increased risk of detection of cyfluthrin (56).

A cohort study analyzed dust samples contained in vehicles and homes and found higher DAP levels in urine samples in resident children (*n* = 170) than in homes of farm workers exposed to occupational organophosphates. Mean concentrations of AZM, CPS, ML, and PH in households inhabited by a farm worker exceeded the levels of those households where a farmer does not live (57). Weak or non-significant associations were related to increased DAP levels with an elevated household income, along with members of a household where someone works with pesticides, including those who live on a farm or drink water from an open source, or eat from a vineyard or garden crops (44).

#### 3.2.3. Educational interventions

Regarding educational intervention, researchers reported that it was not associated with a reduction in urinary metabolite levels, nor were there any significant differences between the pre- and post-measures (58). In Italy, Bravo et al. found an association between greater parental education and higher concentrations of OPs and PYR metabolites, based on a cohort design on urine samples of the infant population (*n* = 199, 7 years of age), which may reflect different eating habits, however, fish consumption is not related to concentrations of POs and PYR (59).

González-Alzaga et al. identified that the number of years of formal education of the mother and the variables related to the residential environment and exposures at home are the most important determinants for the presence of DAP metabolites (35).

Cartier et al. found no evidence that prenatal OP exposure adversely affected cognitive function in 6-year-olds, perhaps because of the population's socioeconomic status, which was higher than in previous studies, though other causal and non-causal explanations are also possible (60).

### 3.3. Consequences on children and teenagers

Some authors suggest potential associations of chlorophenol pesticides with being overweight, obese, and a lipid profile, along with blood pressure in children and adolescents (61) and adverse neurodevelopmental effects associated with early childhood CPS exposure, but not prenatal exposure (62). The compound 3-PBA in children's urine samples was positively associated with BMI z-scores, however, its concentrations at midterm pregnancy reported no association (63). Pyrethroid and organophosphate pesticide exposure might have harmful effects on children's verbal and memory development (64). Researchers reported associations related to pyrethroid insecticide use and urinary 3-PBA concentrations among preschool-age boys (65). Neuropsychological impairment was reported as a negative effect after pesticide exposure in children, however, no relationship was reported after prenatal exposure in newborns (66).

Oya et al. examined levels of OP pesticides in Japanese toddlers. Some exposure behaviors such as the use of insect repellent sprays, herbicides, and insecticides, were associated with increased creatinine-unadjusted DAP concentrations (67).

In Japan, Yoshida et al. found that the main route of exposure for DCB (dichlorobenzene) absorption in children was considered to be inhalation while at home. Indoor concentrations of dichlorobenzene surpassed the risk of cancer over a lifetime from 10^−3^ in 9% of the residences and 10^−4^ in 22% of them (68).

Suh et al. demonstrated no relationship between agricultural pesticides and the development of precocious puberty (69). Van Wendel de Joode et al. found that after adjustment for potential confounders, higher urinary TCPy concentrations were associated with poorer working memory in boys, poorer visuomotor coordination, and increased prevalence of parent-reported cognitive problems/inattention due to children living near banana plantations who were exposed to pesticides that may affect their neurodevelopment (70). Zhang et al. demonstrated that children in an agricultural region of China were exposed to carbamate pesticides. Exposure *in utero* and at 3 and 7 years old may adversely impact a child's neurodevelopment (71). Fiedler et al. found that no significant adverse neurobehavioral effects were observed between participant groups during either the high or low pesticide use season, however, due to the small sample size, any significant differences observed should be regarded with caution (72).

Neurodevelopmental effects were reported in Chinese children from agricultural communities that were exposed (pre and postnatal) to carbamate pesticides (73). Poorer Verbal IQ in boys was associated with maternal urinary DEAPs but showed no effects in girls (74). Organophosphate exposure (pyrethroids) has been associated with attention-deficit/hyperactivity disorder frequency (75), the elevation of gonadotropin serum levels, and early puberty in boys (76). Thyroid gland diseases have been related to organophosphate exposure. The prevalence of hypothyroidism was reported as higher in children with positive urinary metabolites. A study reported pesticide exposure as a risk factor in children living in rural areas (77). Kidney injury or renal damage has been studied by several authors. Some have found no evidence or association between renal injury in children exposed to low levels of glyphosate (78). Other reported weak evidence which suggests that urinary DAP metabolites are related to kidney injury among children with CKD and poor outcomes (79).

DNA damage was reported in children exposed to aromatic hydrocarbons and DDT compared to samples from children living in low-exposure areas (80). Other organ damage such as pulmonary and brain diseases have been reported and suggested to be secondary to PYR environmental exposure in teenagers (lower forced expiratory flow/forced vital capacity). Furthermore, increased frequency of cytogenetic damage markers, delayed puberty onset in girls, and enhanced risk of neurodevelopmental diseases have been reported (81–87). A cohort study in the USA, in an infant population (*n* = 279, 6–60 months of age), determined that total urinary concentrations of dialkyl phosphate (OP) were associated with a significant decrease in lung function in children aged 7 years (88). Another study evaluated differences between different contaminants in children urine (*n* = 226, 6–11 years of age). It found no differences for any PP (priority pesticides), and BMI was negatively associated with OPP levels, while in girls higher levels of PAH, EPH, and PPs metabolites were found than in children, adolescents, adults, and older adults (89). In Thailand, Sapbamrer et al. determined that DAP (dialkyl phosphate) levels were significantly higher in children living in farming communities than those living in urban communities. This study suggests that children may be exposed to OPs both inside and outside the home and that this exposure can cause oxidative stress in children. Oxidative stress contributes to the development of chronic diseases and is recommended to be measured as a biomarker among children exposed to POs as a preventive way to identify chronic diseases (90).

Oxidative damage is one of the most commonly studied effects. DNA can be damaged by oxidative stress which has several consequences. In 2021, Konstantinuo et al. presented a metabolomics study that showed a reduction in biomarkers of oxidative stress in children after a dietary intervention. Reducing the exposure to pyrethroids and neonicotinoids resulted in better metabolism of glucose and uric acid (91).

Glyphosate is a common compound used around the world. Some compounds can be found in the air, soil, water, and meals (92). Exposed children can suffer respiratory symptoms (cough and bronchospasm), and skin damage. DNA damage has been demonstrated in children who are exposed to glyphosate. Genetic damage observed in micronucleus assays was a real warning of its potentially harmful effects on health (93). As hormone-disruptive agents (xenobiotics), pesticides were associated with an increased prevalence of cryptorchidism in areas of Europe where the amounts of these compounds were higher (94).

As previously mentioned, pesticides can trigger mutagenic processes in different pathways. DNA damage observed in micronucleus tests has been reported in populations that have been exposed to pesticides compared to those in non-exposed groups. However, there was no definitive conclusion regarding the future results of DNA damage since a comparison of biomarkers between groups showed no differences (95).

Other studies have reported an increased risk of cancer and respiratory problems in children (15, 87). Similarly, neurological and behavioral changes in children have been associated with pesticide exposure. A specific mechanism is unclear, but the main theory is that it is induced by oxidative stress and DNA damage (73, 74, 76, 96).

Several authors have studied the relationship between kidney damage and pesticide exposure (78, 79). Evidence of this relationship is weak. Studies have reported no association between glyphosate levels and albuminuria in regions with the highest incidence reports of chronic renal disease (42, 97).

Research regarding genotoxic risk among children exposed to pesticides concluded that there was a significant positive association between the extent of DNA damage and the age of the children, length of residence in the area, pesticide detection, and frequency of apple consumption (98).

In 2017, Weldon et al. reported changes in miRNAs related to total urinary concentration of DAPs, (major metabolites of organophosphates pesticides) in farmworkers' children during the post-harvest season compared with the thinning season (75). Other studies reported a decrease in DAP metabolite levels (51%) after an educational home intervention (96). Pyrethroid pesticide exposure and ADHD association were reported as having increased and were the cause for hyperactive-impulsive symptoms in children aged 8–11 years old and had deleterious effects on the children's neurodevelopment (99, 100). Fluegge et al. found that prenatal PYRs exposure exerts heterogeneous effects by class on mental, but not motor, functioning at 3 months of age (101).

Exposure to certain pyrethroids (PBA, DCCA) at environmental levels may negatively affect neurobehavioral development by 6 years of age (102). These same authors reported that low-level childhood exposures to deltamethrin (cis-DBCA), in particular, and for pyrethroid insecticides, in general (as reflected in levels of the 3-PBA metabolite) may negatively affect neurocognitive development by 6 years of age (103).

In general terms, the amount of reported information regarding health damage is increasing. However, currently, no cause-effect fact has been reported between a specific pesticide and a specific disease. Available research assumes certain facts regarding metabolic pathways which are affected by DNA damage and the resulting consequences. International scientific information has not been good enough to change public policy regarding the indiscriminate use of pesticides.

### 3.4. Measuring pesticides

In 2018, a cohort study performed in Europe determined that for the quantification of the variability of non-persistent chemicals in urine, approximately a dozen samples were required to accurately assess exposure for periods spanning several quarters or 1 month (104). Hyland et al. determined that risk assessments of pesticide exposure from the analysis of urine samples that are not taken immediately in the morning may underestimate the daily dose of organophosphates (OP). They recommend collection and analysis of the first-morning spot samples to more accurately characterize the concentration of OP pesticides in children (105). Hernández AF et al. found that human hair has advantages over urine samples for the evaluation of cumulative exposure to organophosphates. This resulted from analyzing data from the child population in Almeria, Spain (*n* = 222, average age 7.5 years), and residents in the vicinity of areas of intensive agricultural use of greenhouses (106).

Calafat et al. analyzed the feasibility of urine collection for the determination of various substances. They recommend the urine sample as a biomarker for evaluation of exposure to these substances in 3–5 year old children (107). These same authors focused on evaluating the level of exposure to DEET in the general population (*n* = 5,348, 6>years of age). It was found that the highest concentrations of DCBA were greater in the period from May to September than in the October-April period. However, they noted that the general US population, including school-age children, is exposed to DEET, yet it is not advisable to rely solely on the presence of DEET as the only urinary biomarker as it could probably underestimate the prevalence of exposure (108).

A case-control study performed in Japan determined an association between the detection of urinary concentrations and the prevalence of N-desmethyl acetamiprid and neonicotinoid symptoms (109). It was also identified that urine samples collected during the summer were more susceptible to having concentrations of metabolites above the 95th percentile than those collected during the winter (48). In 2021, research carried out in Mexico showed that urinary glyphosate concentrations might vary depending on the season, due to consumption habits and the ready availability of products (110). A cohort study in Japan characterized childhood exposure to NEOs, OPs, and PYRs in 3-year-old children (*n* = 223), in which they found that urinary concentrations of NEO and PYR metabolites were significantly higher in the summer than in winter, meaning that children in Japan are environmentally exposed to three major lines of insecticides and that daily sources of exposure to NEOs are more common than those of OPs (111).

In another Japanese cohort study, urine samples from children *n* = 150 (3 years of age) were analyzed to look for differences in seasonal concentration levels. The results showed that in Japanese children, exposure to PYR-related hygiene products has increased in the past decade (2006–2015), while exposure to higher levels of hygiene-PYR occurs more in the summer than in the winter (112). Ueyama J. et al. established a method with high detection ranges for the biomonitoring of neonicotinoids (NEOs), dinotefuran, and N-desmethyl acetamiprid in urine extracted from disposable diapers in Japan (113). Hernández et *al*. measured concentrations of six OP metabolites in serum and urine in children and adolescents *n* = 60 (average age of 11.8 years) and determined that the levels found of OC and OP lend credibility to the national estimates in the US (Centers for Disease Control and Prevention, Fourth report on human exposure), so these values can be considered as a baseline for children and adolescents of Mexican descent residing in the Lower Rio Grande Valley (114).

## 4. Discussion

Pesticides are a mix of ingredients and substances used to eliminate or control unwanted vegetable or animal species that are recognized as plagues. Pesticides are categorized according to their target pest. In general, there are herbicides for weeds and vegetation, insecticides for insects, and fungicides for fungi (50). People are exposed to pesticide residues through fruit and vegetable intake (20, 21, 24–26, 29, 32), as well as the environment and household insecticide use (32–34, 36–40, 82, 84, 98).

Long-standing use and the absence or inadequate practices of protective equipment imply pesticide exposures, mainly in farmworker housing complexes and through agriculture-related activities. Pesticide exposure has been studied due to public health concerns, particularly in populations with children. Neurobehavioral damage (68), serum glucose alterations, obesity (62), and harmful effects on pulmonary function (88) are health outcomes that have been studied. Gas and liquid chromatography-tandem mass spectrometry analysis methods have been used predominantly for metabolite-pesticide detection in urine samples (24, 30, 33, 34, 36, 44, 83, 85).

The 24-h urine samples are considered the “gold standard” for evaluating daily exposure to pesticides and other environmental chemicals that are excreted in the urine. However, non-first-morning spot samples might underestimate the daily organophosphate dose and the percentage of children with concentrations that exceeds the regulatory levels. First-morning spot samples are recommended for characterizing the OP dose more accurately in children (23). However, some authors consider that hair samples are the best way for performing studies that measure long-term pesticide exposure (36).

In Mexico, urinary glyphosate herbicide was detected in 100 and 73% of children in two different communities. In addition, 17 other pesticides were present, which reveals that there are important exposure circumstances, thus strategies aimed to decrease those levels are needed (42). Common urinary organophosphate metabolites (OP) are DETP, followed by DMTP and DEP (32).

Information regarding pesticides and their interaction with the environment and human health is extensive. A search in PubMed using the words “pesticide” and “health” yielded around 40,000 hints. The most important limitation of the present study was to delimit and select the most relevant information. Around 20 years ago, several articles reported assumptions concerning the harmful effects of pesticides on children. Moreover, new information reports facts with a sustainable methodology. As a scoping review, the main objective of this study was to provide readers with a general panorama of the topic (20), which might be considered as the main limitation. Trying to compile all the available information would be ideal for a book that covers this topic. The strengths of this article lie in examining the scope and nature of the evidence on the subject. After reading the information, readers will be able to make their own assumptions. The authors did not assume any relationship between a pesticide and a disease.

## 5. Conclusion

International evidence in PubMed supports that organic diets in children are successful interventions that can mainly decrease the urinary levels of pesticides, insecticides, and herbicides. First-morning spot samples are the most recommended for accurately characterizing an OP dose in children. Neurobehavioral damage-targeted studies were predominant, however, more studies on organ damage associated with urinary pesticides and cohort studies are needed to understand health damage to children. Only a few urinary pesticide-related studies were found in Mexico and Latin America, therefore, there is a knowledge gap that must be attended to, since agricultural and farming activities are predominant and extend through the respective territories.

## Author contributions

HG-T, AR-d-A, and ES-D: Conceptualization, project administration, and writing–original draft preparation. ES-P, RC, AR-d-A, MG-G, and FL-K: Investigation and writing–review. ES-D: Editing. All authors have read and agreed to the published version of the manuscript, participated directly in the manuscript, the investigation, writing the original draft preparation, and the writing of the review.
