# Supplementary material for: Frequency of urinary pesticides in children: a scoping review
Source: Front Public Health. 2023 Aug 29;11:1227337. doi: 10.3389/fpubh.2023.1227337 (PMC10497881; doi:10.3389/fpubh.2023.1227337)
Supplement: Supplementary file 1 [file Table_1.pdf]

Supplementary Table 1. Characteristics and main findings of the studies included

Author (year), Country	Study Design	Sample (age)	Urine Pesticide analysis method	Results
Li Y., (2019) Australia [23]	Cross-sectional	n=400 (0-5 years)	GC-MS/MS	Authors found a significant increase in the concentrations of DETP, TCPY, 4-nitrophenol and 3-PBA according to age, suggesting that exposure increases after weaning or as a result of increased dietary intake, as well as mobility and activity
Myridakis A., (2016) Greece [24]	Cross-sectional	n=500 (4.2 years mean)	High-pressure liquid chromatography combined with electrospray ionization tandem mass spectrometry	The presence of organophosphate levels is linked to food consumption.
Sinha S. N., (2018) India [25]	Cross-sectional	n=377 (6-15 years)	LCMS/ MS	Authors found that on a conventional diet, girls have 87% higher levels of DAP pesticides than Boys.
Hyland, C., (2019). USA [26]	Cohort	n= 9 (4-15years)	Liquid-chromatography-tandem mass spectrometry (LC-MS/MS)	Organic diet interventions significantly decreased urine levels of 13 pesticide metabolites (organophosphates)
Fagan (2020) USA. [27]	Cohort	n=9 (4-15 years)	Shimadzu Nexera X2 ultra high-performance liquid chromatograph.	The 6-day intervention based on an organic diet
Bradman (2015) USA [28]	Cross-sectional	n=40 (3-6 years)	By isotope dilution gas chromatography tandem mass spectrometry	Organic diet decreases concentrations of metabolites in organophosphate insecticides and the herbicide 2,4-D in urine levels in children

Venners S. A., (2017) Canada [29]	Cross-sectional	n=34 (1.5-5 years)	Solid phase extraction and LC/MS/MS	There was a significant lack of association between pesticides by law and urinary 2-4D, which may be due to the use of a small sample size, while the lack of recent acute exposure or that exposure to this pesticide is primarily influenced by sources of exposure not provided for in the law. Among other contributions, this study points out that food may be a route of exposure to 2,4-D
Papadopoulou, E., (2019). UK, France, Spain, Lituania, Norway, Greece. [30]	Cross-sectional	n=1288 (6-11 years)	Ultra-high performance liquid chromatography-quadrupole time-of-flight mass spectrometry UHPLC-TOFMS – described in Cequier et al. (2016)	Fruit consumption is positively associated with the presence of organophosphate pesticide metabolites in infant urine. Consumption of organic foods is negatively associated with organophosphate metabolites
Cequier E., (2017) Norway [31]	Cohort	n=54 (6-12 years)	Ion-pair chromatography	Fruit consumption is the largest factor contributing to variation in urine DAP levels.
Lemke N., (2021) Germany [32]	Cross-sectional	n=2144 (3-17 years)	Gas chromatography coupled to tandem mass spectrometry (GC–MS/MS)	There is no clear association between exposure to glyphosate or AMPA and vegetarian diet or consumption of cereals, pulses, or vegetables, nor could it be identified
Holme F., (2016) USA [33]	Cohort	n=194 (1-7 years)	High performance liquid chromatography-linked tandem mass spectrometry (HPLC-MS/MS).	Significant relationships were found between vegetable consumption during the harvest season and the presence of levels of the metabolite dimethyl.
Berman T., (2020) Israel [34]	Cross-sectional	n=103 (4-11 years)	GC–MS/MS	Fruit consumption is associated with higher levels of DAP metabolites in urine

Mat Sutris J., (2016) Malaysia [35]	Cross-sectional	n=180 (7-12 years)	Gas chromatographyemass spectrometry	Eighty-four (46.7%) of the respondents were positive for urine dialkyl phosphate metabolites. In multivariable analysis, children who frequently consumed apples had 4 times higher risk of pesticide detection than those who consumed apple less frequently. In addition, those who frequently ate cucumbers had 4 times higher risk for pesticide detection than those who ate cucumbers less frequently. Children with a father whose occupation involved high exposure to pesticides (agriculture) had 3 times higher risk of pesticide detection than those with a father in a low-risk occupation
Gonzalez-Alzaga B., (2020) Spain [36]	Cohort	n=559 (3-11 years)	UHPLC-QqQ-MS/MS	The number of years of formal education of the mother and the variables related to the residential environment and exposures at home are the most important determinants for the presence of DAP metabolites, while in terms of diet, banana consumption and not washing fruit before consumption are determinants of exposure levels.
English K., (2019) Australia [37]	Cross-sectional	n=56 (12.9 months mean)	TRACE GC Ultra coupled to a TSQ Quantum XLS triple quadrupole mass spectrometer equipped with a TriPlus Autosampler. UHPLC system, coupled with a tandem mass spectrometer equipped with an IonDrive source.	Factors as diet, age, mobility, having pets, frequency of pesticide use at home, frequency of hand washing and season, are associated with presence of insecticide urinary metabolites

Thomas M. B., (2017) USA [38]	Cross-sectional	n=41 (15-18 months)	Negative electrospray ionization liquid chromatography tandem mass spectometry	Diet is not significantly associated with urine metabolite concentrations OPFR (organophosphate flame retardants), while meat and fish consumption may be associated with higher levels of DPP (Diphenyl phosphate) and BDCPP. Increase in the consumption of dairy and fresh food decreases these levels.
Galea K. S., (2015) UK [39]	Cross-sectional	n=24 (4-12 years)	Liquid chromatography triple quadrupole mass spectrometry for chlormequat; Liquid chromatography-tandem mass spectrometry (LC/APCI-MS/MS) methods for the quantification of captan;	Over 98% and 97% of the measured urinary biomarker concentrations for penconazole and captan respectively were lower than the REA-predicted exposures. Although a number of the chlorpyrifos and chlormequat spray-related urinary biomarker concentrations were greater than the predictions, investigation of the background urinary biomarker concentrations suggests these were not significantly different from the levels expected had no pesticide spraying occurred. The majority of measured concentrations being well below the REA-predicted concentrations indicate that, in these cases, the REA is sufficiently conservative.
Werthmann D. W., (2021) USA [40]	Cohort	n=68 (9.6 years mean)	Semi-automated solid phase extraction method coupled with isotope dilution for the mass spectrometric quantification	The green building practices had no impact on children´s pyrethroid urinary concentrations.
Ikenaka Y., (2019) Japan [41]	Cross-sectional	n=46 (3-6 years)	LC-ESI/MS/MS	Childhood exposure to neonicotinoid pesticides by inhalation is not associated with the season of application. The presence of 6 neonicotinoids reflects the high intake of agricultural products

Heffernan A. L., (2016) Australia [42]	Cross-sectional	n=100 (0-60 years)	Solid-phase extraction coupled with isotope dilution high-performance liquid chromatography–tandem mass spectrometry	The highest concentrations of 5 organophosphate metabolites were found in the youngest and oldest strata. This may be related to age-specific differences such as behavior or physiology. Additionally, it was found that the levels of metabolites of organophosphate insecticides chlorpyrifos were higher than those reported in the US and Canada. This may be due to differences in the registered applications of pesticides that exist between countries
Sierra-Diaz E., (2019) Mexico [43]	Cross-sectional	Group 1: n= 192 (5-15 years) Group 2: n=89 (5-15 years)	HPLC/MS/MS (high-performance liquid chromatography coupled with tandem mass spectrometry	A total of 17 pesticides were found in 100% of urine samples from children from two agricultural communities.
Bravo N., (2020) Slovenia [44]	Cross-sectional	n=168 (7-8 years)	Ultra-Performance Liquid Chromatography (UPLC Acquity H-Class	The highest concentrations of all metabolites of organophosphate pesticides (PNP, 3-PBA) and pyrethroids (TCPY) analyzed in urine samples were found in children in comparison with their mothers.
Raherison C., (2019) France [45]	Cohort	n=281 (7.5 years mean)	Liquid chromatography coupled with tandem mass spectrometry (LC-MS/MS)	Children living in rural areas near vineyards are at increased risk of exposure to dithiocarbamates during the summer, while an association was found between urinary concentrations of ETU and symptoms of asthma and rhinitis.
Muñoz-Quezada M. T., (2020) Chile [46]	Cohort	n=48 (mean age 9 years)	Reversed-phase high performance liquid chromatography technique, and detected by using tandem mass spectrometry with	Authors found that all the children Living near farms that were evaluated had more than two metabolites of urinary pesticides, where 3-PBA was the most frequent

			isotope dilution quantitation	
Molomo R. N., (2021) South Africa [47]	Cross-sectional	n=183 (5-19 years)	Liquid chromatography with tandem mass spectrometer detection (LC-MS/MS	DAP concentrations were lower in the grape and wheat planting area than in those of pepita. The study further notes that younger children living near grape and apple farms are associated with increased urinary DAP concentrations. Weak or non-significant associations were related to increased DAP levels with an elevated household income, long with members of a household where someone works with pesticides, including those who live on a farm or drink water from an open source or eat from a vineyard or garden crops.
Jain R. B., (2016) USA [48]	Cross-sectional	n= 2830 (6-19 years)	Isotope-dilution gas chromatography–tandem mass spectrometry (GC–MS/MS).	Exposure both inside and outside the home does not affect the levels of any dialkylphosphates. The group of children always presented higher levels than adolescents and adults.
Yoshida T., (2021) Japan [49]	Cross-sectional	n=132 (6-15 years)	Gas chromatograph mass spectrometer	The analysis of urinary pesticides and air quality within walls, showed that transfluthrin was the most remarkable pyrethroid as an intramural pollutant.
Tao Y., (2019) China [50]	Cross-sectional	n=247 (4.8 years mean)	LC-MS/MS	Younger children tend to be at higher risk of exposure when living in rural areas. Inhabitants of areas with orchids were more exposed to IMI to various degrees.

Ospina M., (2019) USA [51]	Cross-sectional	n=3038 (3-6 years)	Reversed phase high-performance liquid chromatography	Asian children group was more likely than non-Asians to have MAPD concentrations greater than the 95th percentile. It was also identified that urine samples collected during the summer were more susceptible to having concentrations of metabolites above the 95th percentile than those collected during the winter.
Song W., (2021) China [52]	Cross-sectional	n=108 (0-7 years)	LC-MS/MS	Authors found a positive correlation between 2,4-D concentrations and the biomarker of oxidative stress 8-OHdG in young children.
Jo HM., (2015) South Korea [53]	Cross-sectional	n=70 (6-12 years)	PerkinElmer Clarus 600T GC-MS	All respondents had values above the detection limit, and the geometric means of 3-PBA in all children were 1.85 µg/L and 1.46 µg/g creatinine. Children with the top 10% urinary levels of 3-PBA were more likely to be girls, under nine years of age, living in a rural area, and living in a residential type apartment.
Lehmler H. J., (2020) USA [54]	Cross-sectional	n=2295 (6-19 years)	Liquid chromatography-tandem mass spectrometry.	The age, gender, race/ethnicity and PIR were associated with levels of 3-PBA.
Galea K. S., (2015) UK [55]	Cross-sectional	n=22 (4-12 years)	Liquid chromatography triple quadrupole mass spectrometry for chlormequat; Liquid chromatography-tandem mass spectrometry (LC/APCI-MS/MS) methods for the quantification of captan;	The levels of penconazole and captan were lower than the exposures predicted by REA, however, background urinary biomarkers suggest that chlorpyrifos and chlormequat (associated with pesticide application events) are not significantly different from the expected levels when no app.

Li A. J., (2018) USA, Greece, China, India, Saudi Arabia, Japan, Korea and Vietnam [56]	Cohort	n=322 (1-83 years)	High-performance liquid chromatography-tandem mass spectrometry (HPLC-MS/MS).	The intake of chlorpyrifos is higher in populations of Vietnam, Greece, India, China and Korea (≥9.6µg/day) than those estimated for other countries (<5µg/day). Similarly, the daily intake of parathion was found to be higher in China, India, and Korea, than estimated in other countries (5.7-9.3µg/day)
Glorennec P., (2017) France [57]	Cohort	n=245 (6 years)	Ultra-performance liquid chromatography and triple quadrupole mass spectrometry. Cis-DCCA	Children with a parent occupationally exposed to pesticides were about 3-times more likely to have higher urinary concentrations of 3-PBA (OR = 2.8, 95% CI [1.2; 6.5]). Dust content was correlated mainly with household insecticide use: higher mean concentrations of permethrin (beta = 0.8 [0.3; 1.3], in mu g/g) and an increased risk of a detectable level of cyfluthrin (OR = 4.7 [1.7; 12.9]) were observed in home dust, for indoor use of at least twice a year. Outdoor insecticide use at least once a year was associated with detection in dust of cypermethrin (OR = 3.0 [1.3; 6.7]) and tetramethrin (OR = 3.7 [1.6; 8.3]).
Tamaro CM., (2018) USA [58]	Cohort	n=170 (2-6 years)	Gas chromatography-mass spectrometry and isotope dilution quantification	Higher DAP levels in urine samples were found in children whose father is farmworker exposed to OP.
Muñoz-Quezada M. T., (2019) Chile [59]	Case Control	Control n= 22 Intervention n= 26 (7-11 years both)	Reversed-phase high-performance liquid chromatography technique	The educational intervention, is not associated with a reduction in urinary metabolites levels, nor were there any significant differences between the pre and post measures.

Bravo N., (2019) Italy [60]	Cohort	n=199 (7 years old)	Isotope dilution solid phase extraction UPLC-MS/MS using an Ultra-P erformance Liquid Chromatography	Authors found an association between greater parental education and higher concentrations of Ops and PYR metabolites, based on a cohort design on urine samples of the infant population, which may reflect different eating habits. Fish consumption is not related to concentrations of OPs and PYR.
Cartier (2016) France [61]	Cross-sectional	n=231 (6 years)	LC/MS-MS	Researchers found no evidence that prenatal OP exposure adversely affected cognitive function in 6-year-olds, perhaps because of the population's socioeconomic status, which was higher than in previous studies, though other causal and non-causal explanations are also possible.
Parastar S., (2018) Iran [62]	Cross-sectional	n= 242 (6-18 years)	Mass spectrometric measurement was performed in electron ionization mode.	There are potential associations of chlorophenol pesticides with being overweight, obese, and a lipid profile along with blood pressure in children and adolescents.
Guo J., (2019) China [63]	Cohort	n=377 (3 years)	Large-volume-injection gas chromatography tandem mass spectrometry (LVI-GC-MS/MS)	Adverse neurodevelopmental effects are associated with early childhood CPF exposure, but not prenatal exposure.
Lee K. S., (2019) South Korea [64]	Cohort	n=578 (4 years)	GCMS/MS	The compound 3-PBA in children's urine samples was positively associated with BMI z-scores.
Wang N., (2016) China [65]	Cross-sectional	n=406 (3-6 years)	GC-MS-MS	Pyrethroid and organophosphate pesticide exposure might have harmful effects on children verbal and memory development.

Lee W. S., (2020) South Korea [66]	Cross-sectional	n=385 (4 years)	Gas chromatograph-mass spectrometer	Researchers reported associations related to pyrethroid insecticide use and urinary 3-PBA concentrations among preschool-age boys.
Gonzalez-Alzaga (2015) [67]	Ambispective	n=305 (6-11 years)	GC–MS/MS	Neuropsychological impairment was reported as a negative effect after pesticide exposure in children, however, no relationship was reported after prenatal exposure in newborns
Oya N., (2020) Japan [68]	Cohort	n=1037 (0-40 months)	High-performance liquid chromatography with tandem mass spectrometry (LC-MS/MS).	Some exposure behaviors such as use of insect repellent sprays, herbicides and insecticides, were associated with increased Creatinine-unadjusted DAP concentrations.
Yoshida T., (2021) Japan [69]	Cross sectional	n=112 (6-15 years)	Gas chromatography/mass spectrometry (GC/MS).	The main route of exposure for DCB (Dichlorobenzene) absorption in children was considered to be inhalation while at home. Indoor concentrations of Dichlorobenzene overpassed the lifetime cancer risk level of 10^-3 in 9% of the residences and 10^-4 in 22% of them.
Suh. J., (2020) South Korea [70]	Case control	case n=30 control n=30 (8.5 years mean)	LC-MS/MS	Authors demonstrated no relationship between agricultural pesticides and the development of precocious puberty.
Van Wendel de Joode B., (2016) Costa Rica [71]	Cross-sectional	n=140 (6-9 years)	LC-MS/MS	Researchers found that after adjustment for potential cofounders, higher urinary TCPy concentrations were associated with poorer working memory in boys, poorer visuo-motor coordination, and increased prevalence of parent reported cognitive problems/inattention due to children living near banana plantations who are

				exposed to pesticides that may affect their neurodevelopment.
Zhang J., (2020) China [72]	Cohort	n=303 (3-7 years)	Gas chromatography tandem mass spectrometry (GC-MS/MS)	Exposure in utero and at three and seven years may adversely impact a child´s neurodevelopment.
Fiedler N., (2015) Thailand [73]	Case Control	Case n=25 Control=29 (6-8 years)	Gas chromatograph with tandem mass spectrometric analysis	No significant adverse neurobehavioral effects were observed between participant groups during either the high or low pesticide use season, however, due to the small sample size, any significant differences observed should be regarded with caution.
Zhang J., (2019) China [74]	Cohort	n=377 (3 years mean)	GC-MS/MS	Neurodevelopmental effects were reported in Chinese children from agricultural communities, exposed (pre and postnatal) to carbamate pesticides.
Ntantu Nkisa P., (2020) Canada [75]	Cross-sectional	n=607 (3-4 years)	Gas chromatography coupled with mass spectrometry	Poorer Verbal IQ in boys was associated with maternal urinary DEAPs and no effects in girls.
Weldon B. A., (2016) USA [76]	Cross-sectional	n=16 (6-10 years)	HPLC-MS/MS	There is positive association between miRNAs and urinary pesticides in children while postharvest season.
Chang C.H., (2021) [77]	Case control	Case n= 85 Control n=96 (9 years mean)	Gas chromatography–mass spectrometry (GC–MS).	Organophosphate exposure (pyretroid) has been associated to attention-deficit/hyperactivity disorder frequency.
Ye X., (2017) China [78]	Cross-sectional	n=463 (9-16 years)	Ultra-performance liquid chromatography−triple quadrupole mass spectrometry	This work reports on an association of increased pyrethroid exposure with elevated gonadotropins levels and earlier pubertal development in boys.

Suhartono S., (2018) India [79]	Cross sectional	n=66 (9.2 years mean)	Isotope-dilution gas chromatography-tandem mass spectrometry.	Thyroid gland diseases have been related to organophosphate exposure. Prevalence of hypothyroidism was reported as higher in children with positive urinary metabolites. The study reported as risk factor the pesticide exposure in children living in rural areas.
Trasande L., USA (2020) [80]	Cross-sectional	n=108 (10-19 months)	LC-MS/MS	Some authors have found no evidence or association between renal injury in children exposed to low levels of glyphosate.
Jacobson M. H., (2021) USA-Canada [81]	Cohort	n=618 (6 months to 6 years)	Mass spectrometric analysis	There is a weak evidence suggesting that urinary DAP metabolites are related to kidney injury among children with CKD, and poor outcomes.
Jasso-Pineda Y., (2015) Mexico [82]	Cross-sectional	n=256 (6-12 years)	HPLC	DNA damage was reported in children exposed to aromatic hydrocarbons and DDT compared to samples from children living in low exposure areas.
Hu P., (2021) USA [83]	Cross-sectional	n=1174 (6-17 years)	High performance liquid chromatography/tandem mass spectrometry	Environmental PYR exposures may adversely affect children’s pulmonary function, with the strongest associations among 11–17 years old boys.
Chen S., (2016) China [84]	Case control	Case n=161 Control n= 170 (0-14 years)	Gas chromatography-mass spectrometry (GC-MS)	The exposure to pyrethroid pesticides might be associated with increased risk of CBT (Childhood brain tumors).
Ye M., (2016) Canada [85]	Cross-sectional	n=5436 (6-79 years)	Gas chromatography-mass spectrometry	The pyrethroid exposures were associated with lower FEV1 in children, lower FVC in adolescents and relatively higher FEV1/FVC ratio in adults. Forced Vital capacity.

Ruiz-Guzman J. A., (2017) [86]	Case control	Case n= 50<br>Control n=13	Gas chromatography-mass spectrometry	Infant exposure to one of the more-often used pesticides (atrazine and its metabolites) in the agricultural areas evaluated and an increasing trend in the frequency of markers of cytogenetic damage in the groups of the agricultural areas, as compared to the control group, were evident.
Ye X., (2017) China [87]	Cross-sectional	n=305 (9-15 years)	Ultraperformance liquid chromatography-triple quadrupole mass spectrometry	Pyrethroids exposure may increase the risk of delayed pubertal onset in girls.
Waits A., (2021) Taiwan [88]	Case control	Case n=76<br>Control n=98 (4-15 years both)	Ultra-performance liquid chromatography-tandem mass spectrometry	The dose-response relationship suggests enhanced susceptibility to EDC burden in children even at lower levels, whereas the main risk is likely from organophosphate pesticides. HNE-MA is recommended as a sensitive biomarker of lipid peroxidation in the further elucidation of the oxidative stress role in ADHD etiology.
Raanan R., (2016) USA [89]	Cohort	n=279 (6-60 months)	Gas chromatography-tandem mass spectrometry and quantified using isotope dilution calibration	Total urinary concentrations of dialkylphosphate (OP) are associated with a significant decrease in lung function in infants aged 7 years.
Jain R.B (2018) USA [90]	Cross-sectional	n=1059 (6-19 years)	On-line solid phase extraction (SPE) coupled to HPLC and tandem mass spectrometry (HPL/CMS/MS).	Authors found no differences for any PP (priority pesticides), and BMI was negatively associated with OPP levels, while in girls higher levels of PAH, EPH and PPs metabolites were found than in children, adolescents, adults, and the elderly.

Sapbamrer R., (2020) Thailand [91]	Cross-sectional	n=161 (7-9 years)	Gas chromatography-flame photometric detector	The DAP (dialkylphosphate) levels were significantly higher in children living in farming communities than those living in urban communities. This study suggests that children may be exposed to OPs both inside and outside the home, and that this exposure can cause oxidative stress in children.
Konstantinou C., (2022) Cyprus [92]	Cohort	n=149 (10-12 years)	GC–MS	D-glucose was associated with the organic food intervention (β = −0.23, 95% CI: −0.37,−0.10), aminomalonic acid showed a time-dependent increase during the intervention period (βint = 0.012; 95% CI:0.002, 0.022) and was associated with the two OD (Oxidative Damage) biomarkers (β = −0.27, 95% CI:−0.34,−0.20 for 8-iso-PGF2a and β = 0.19, 95% CI:0.11,0.28 for 8-OHdG) and uric acid with 8-OHdG (β = 0.19, 95% CI:0.11,0.26). Metabolites were involved in pathways such as the starch and sucrose metabolism and pentose and glucuronate interconversions.
Morgan M., K. (2014) USA [93]	Cross-sectional	n=129 (20-66 months)	Gas chromatograph with mass selective detection	The children's estimated median potential intake doses through dietary ingestion, nondietary ingestion, and inhalation routes were the highest for 2,4-D and cis/trans-permethrin (both 4.84 ng/kg/day), cis/trans-permethrin (2.39 ng/kg/day), and heptachlor (1.71 ng/kg/day), respectively. The children's estimated median potential aggregate intake doses by all three routes were quantifiable for chlorpyrifos (4.6 ng/kg/day), cis/trans-permethrin (12.5 ng/kg/day), and 2,4-D (4.9 ng/kg/day). In conclusion, these children were likely

				exposed daily to several pesticides from several sources and routes at their homes and daycares.
Bernardi N., (2015) Argentina [94]	Cross-sectional	n=75 (9-10 years)	The micronucleus assay was performed on buccal mucosa cells collected using sterile swabs scraped inside the cheeks for 30 seconds, upon rinsing the mouth with drinking water in order to eliminate any food residue. Smears were performed as per the modified method described by Tolbert, et al	A significant difference was observed between exposed children living less than 500 m from areas subjected to spraying and those who were not exposed. Forty percent of exposed children suffer some type of persistent condition, which may be associated with chronic exposure to pesticides

García-Rodríguez J., (1996) Spain [95]	Ecological study	n=126 (1-16 years)	The Agrarian ProtectionAgency, administered by the ProvincialDelegation of the Agricultural Council,Andalusian Regional Government, ratedpesticide use along a 4-point scale from 0(lowest) to 3 (highest) in each municipality	Regression models showed that the strength of association between orchidopexy and level of pesticide use tended to increase with higher levels of use, with the exception of level 0 (mainly in the city of Granada). Our results are compatible with a hypothetical association between exposure to hormone-disruptive chemicals and the induction of cryptorchidism.
Benedetti D., (2018) Brazil [96]		n=137 (44-48 years)	Urine samples was analysed through the Particle-induced X-ray emission (PIXE) technique; Commet Assay were performed to detect DNA damage.	DNA damage could be a consequence of the ability of the complex mixture, including Al and P, to cause oxidative damage. These data indicate that persistent genetic instability associated with hypermethylation of DNA in soybean workers after long-term exposure to a low-level to pesticides mixtures may be critical for the development of adverse health effects such as cancer.
Salvatore A. L., (2015) USA [97]	Case control	Control n=55 case Intervention n=61 (2.2 years mean)	GC-tandem mass spectrometry (GCMS/MS)	Educational home intervention decreased DAP metabolite levels in 51%
Lozano-Kasten (2017) Mexico [98]	Cross-sectional	n=394 (0-17 years)	Two first morning urine samples with an interval of 12 weeks were requested for participants. The samples were analyzed with	The 68.1% of the children studied, were found in stages 3a and 3b of the Kidney Disease Improving Global Outcomes (KDIGO) classification (mean glomerular filtration rate (GFR) 51.9 and 38.4 mL/min/1.73 m2 respectively). The lowest frequencies were for classifications 1 and 4. None of

			reactive strips for the detection of human albumin Micral-Test®, (Roche Diagnostics GmbH, Mannheim, Germany). In those presenting two positive tests for albuminuria, blood tests were carried out for creatinine, urea, and cystatin C Diazyme's Cystatin C Calibrator Set® for immunoturbidometry assay, (Diazyme Laboratories, Inc., San Diego, CA, USA).	the subjects was classified as grade 5. The prevalence of albuminuria in children from this rural community is 3–5 times higher than reported in international literature. Regarding GFR, more than 50% of children studied are under 60 mL/min/1.73 m2. It is a priority to find the causes of albuminuria and CKD in this Mexican region.
Mat Sutris J., (2016) Malaysia [99]	Cross-sectional	n=180 (7-12 years)	Gas chromatography e mass spectrometry	Out of 180 respondents, 84 (46.7%) showed positive traces of organophosphate metabolites in their urine. Children with detectable urinary pesticide had a longer tail length (median 43.5; IQR 30.9 to 68.1 µm) than those with undetectable urinary pesticides (median 24.7; IQR 9.5 to 48.1 µm). There was a significant association between the extent of DNA damage and the children's age, length of residence in the area, pesticides detection, and frequency of apple consumption
Wagner-Schuman M.,	Cross-sectional	n=687 (8-15 years)	High-performance liquid chromatography	Authors found an association between increasing pyrethroid pesticide exposure and ADHD which may be stronger for hyperactive-impulsive symptoms

(2015) USA [100]				compared to inattention and in boys compared to girls.
Yu C. J., (2016) Taiwan [101]	Case control	Case n=97 control n= 110 84-15 years)	Gas chromatography-mass spectrometry (GC-MS).	Children with higher urinary DMP concentrations may have a twofold to threefold increased risk of being diagnosed with ADHD. Organophosphate pesticide exposure may have deleterious effects on children's neurodevelopment, particularly the development of ADHD.
Fluegge K. R., (2016) USA [102]	Cohort	n=118 (2 months age)	GC/MS in the multiple ion detection	Prenatal PYRE exposures exert heterogeneous effects by class on mental, but not motor, functioning at three months of age.
Viel J. F., (2017) France [103]	Cohort	n=281 6 years)	Ultra-performance liquid chromatography and triple quadrupole mass spectrometry (UPLC/MS-MS	Exposure to certain pyrethroids (PBA, DCCA) at environmental levels may negatively affect neurobehavioral development by 6 years of age.
Viel J. F., (2015) France [104]	Cohort	n=281 (6 years)	Ultra-performance liquid chromatography and triple quadrupole mass spectrometry (UPLC/MS-MS	Low-level childhood exposures to deltamethrin (cis-DBCA), in particular, and for pyrethroid insecticides, in general (as reflected in levels of the 3-PBA metabolite) may negatively affect neurocognitive development by 6 years of age.
Casas M., (2018) Spain, France, Olso, UK, Greece, Lithuania [105]	Cohort	n=152 (8 years mean)	Liquid chromatography coupled with mass spectrometry (LC-MS/MS);	About a dozen urine samples are required for the quantification of the variability of non- persistent chemicals in urine, and also for accurately assess exposure for periods spanning several quarters or one month

Hyland C., (2021) USA [106]	Cross-sectional	n=25 (3-6 years)	Gas chromatography-tandem mass spectrometry using isotope dilution	Risk assessments of pesticide exposure from analysis of urine samples that are not taken immediately in the morning may underestimate the daily dose of Organophosphates. Collection and analysis of first morning spot is recommended.
Hernandez A. F., (2019) Spain [107]	Cross sectional	n=222 (7.5 mean age)	Ultra-high performance liquid-chromatography coupled to triple-quadrupole tandem mass-spectrometry (UHPLC-QQQ-MS/MS)	Human hair has advantages over urine samples for the evaluation of cumulative exposure to organophosphates.
Calafat, A. M., (2017) USA [108]	Cross-sectional	n=122 (3-5 years)	Isotope-dilution coupled to mass spectrometry	Urine sample is recommended as biomarker for children evaluation exposure to plasticizers, combustion products, personal care products and pesticides.
Calafat A. M., (2016) USA [109]	Cross-sectional	n=5348 (6 years and older)	Online solid-phase extraction coupled to isotope dilution-high-performance liquid chromatography-tandem mass spectrometry	The highest concentrations of DCBA were higher in the period from May to September than in the October-April period. However, they noted that the general U.S. population, including school-age children, are exposed to DEET, yet it is not advisable to rely solely on the presence of DEET as the only urinary biomarker as it could probably underestimate the prevalence of exposure.
Marfo J. T., (2015) Japan [110]	Case control	Case n=85 (4-87 years) Control n=50	LC/MS/MS	There is an association between the detection of urinary concentrations and the prevalence of N-desmethyl acetamiprid (DMAP) and neonicotin symptoms.

Lozano-Kasten (2021) Mexico [111]	Cross-sectional	n=95 (6-16 years)	High-performance liquid chromatography coupled with tandem mass spectrometry.	All samples tested positive for glyphosate levels. Urine glyphosate levels were related to the season and the age of the children. Glyphosate is present in children of all ages in the community even if they were not in direct contact with it.
Osaka A., (2016) Japan [112]	Cohort	n=223 (3 years)	Modified high performance liquid chromatography-tandem mass spectrometry (LC-MS/MS)	Authors found that urinary concentrations of NEO and PYR metabolites were significantly higher in the summer than in winter, meaning that children in Japan are environmentally exposed to three major lines of insecticides, and that daily sources of exposure to NEOs are common for those of OPs.
Hamada R., (2020) Japan [113]	Cohort	n=150 (3 years)	GCMS/MS	Children exposure to PYR-related hygiene products has increased in the past decade (2006-2015), while exposure to higher levels of hygiene-PYR occur more in the summer than in the winter
Ueyama J., (2020) Japan [114]	Cross-sectional	n=50 (3 year)	LC-MS/MS	Authors established a method with high detection ranges for biomonitoring for neonicotinoids (NEOs), dinotefuran and N-desmethylacetamiprid in urine extracted from disposable diapers in Japan.
Hernandez M., (2019) USA [115]	Cross-sectional	n=60 (5-18 year)	Gas chromatography/tandem mass spectrometry with isotope dilution	The levels found of OC and OP enables the credibility of national estimates in the US (Centers for Disease Control and Prevention, Fourth report on human exposure), so these values can be considered as a baseline for children and adolescents of Mexican descent residing in the Lower Rio Grande Valley.
